# Dual task test: Screening procedure for early detection of cognitive impairment among healthy adults

**DOI:** 10.1093/eurpub/ckac130.055

**Published:** 2022-10-25

**Authors:** H Simi, B Guggenberger, P Holler, M Christova, R Pilz, W Staubmann

**Affiliations:** 1 Sport Science Laboratory, FH JOANNEUM, University of Applied Sciences, Bad Gleichenberg, Austria; 2 Institute of Physiotherapy, FH JOANNEUM, University of Applied Sciences, Graz, Austria; 3 Institute of Dietetics & Nutrition, FH JOANNEUM, University of Applied Sciences, Graz, Austria; 4 Section of Physiology, Otto Loewi Resarch Centre, Medical University of Graz, Graz, Austria; 5 Department of Orthopaedics & Trauma, Medical University of Graz, Graz, Austria

## Abstract

**Background:**

Early detection of cognitive impairment can slow progression to dementia when using appropriate therapy. For early detection of dementia dual task combining cognitive tasks and walking might be suitable, since individuals with cognitive impairment have shown greater changes in gait specific parameters on dual task test (DT) compared to single task test (ST). This study investigates whether these changes correlate with poorer cognitive function in healthy older adults.

**Methods:**

In a cross-sectional study 174 healthy adults (66,48±4,26years; 40%female) completed the Cognitive Functions Dementia Test (CFD), with a lower CFD index indicating lower cognitive function. Participants performed ST (walking 20m) and DT (walking 20m & counting backwards), in which step frequency, stride length and gait speed were monitored by Pablo sensors. Cognitive cost (CC) was determined for each gait variable. CC represents a change score between SD & DT and quantifies cognitive demands, with higher CC indicating poorer cognitive function. Pearson correlations and stepwise linear regression adjusted for age and gender were applied to analyze the association between CFD Index (dependent variable) and CC gate variables (predictors) (α = 5%).

**Results:**

Significant correlations were observed between CFD Index and CC step frequency (p=.014, r=-.187), CC stride length (p=.037, r=-.160) and CC gait speed (p=.002, r=-.232). Since gait variables were intercorrelated (multicollinearity), only gait speed was significant predictor for CFD Index (ß = -.243, p<.001, R^2^ = .053) in a stepwise adjusted regression model.

**Conclusions:**

Changes in gait speed might be sensitive enough to indicate differences of cognitive performance among older individuals. Therefore, DT could be included in screening procedures as alert for potential cognitive decline.

**Key messages:**

- Changes in gait speed might be sensitive enough to indicate differences of cognitive performance among older individuals.
- DT is sensitive to distinguish different cognitive performance in healthy elderly with potential for early detection of mild cognitive impairments.

